# Supplementary material for: Creatine kinase mitochondrial 2 promotes the growth and progression of colorectal cancer via enhancing Warburg effect through lactate dehydrogenase B
Source: PeerJ. 2024 Jun 28;12:e17672. doi: 10.7717/peerj.17672 (PMC11216189; doi:10.7717/peerj.17672)
Supplement: Supplemental Information 3 [file peerj-12-17672-s003.pptx]

## Slide 1
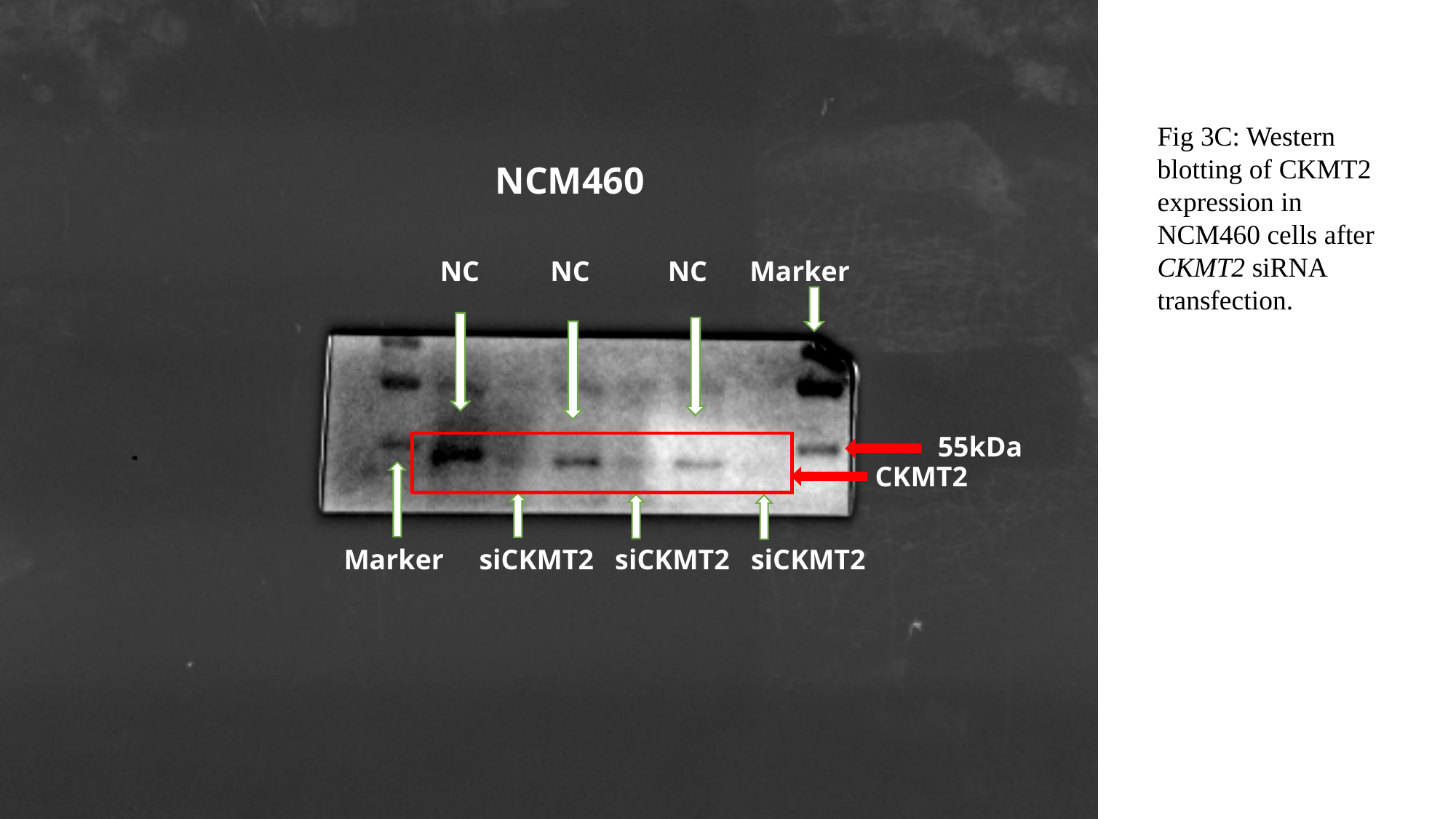

Fig 3C: Western blotting of CKMT2 expression in NCM460 cells after CKMT2 siRNA transfection.
NCM460
 NC NC NC Marker
55kDa
CKMT2
 Marker siCKMT2 siCKMT2 siCKMT2

## Slide 2
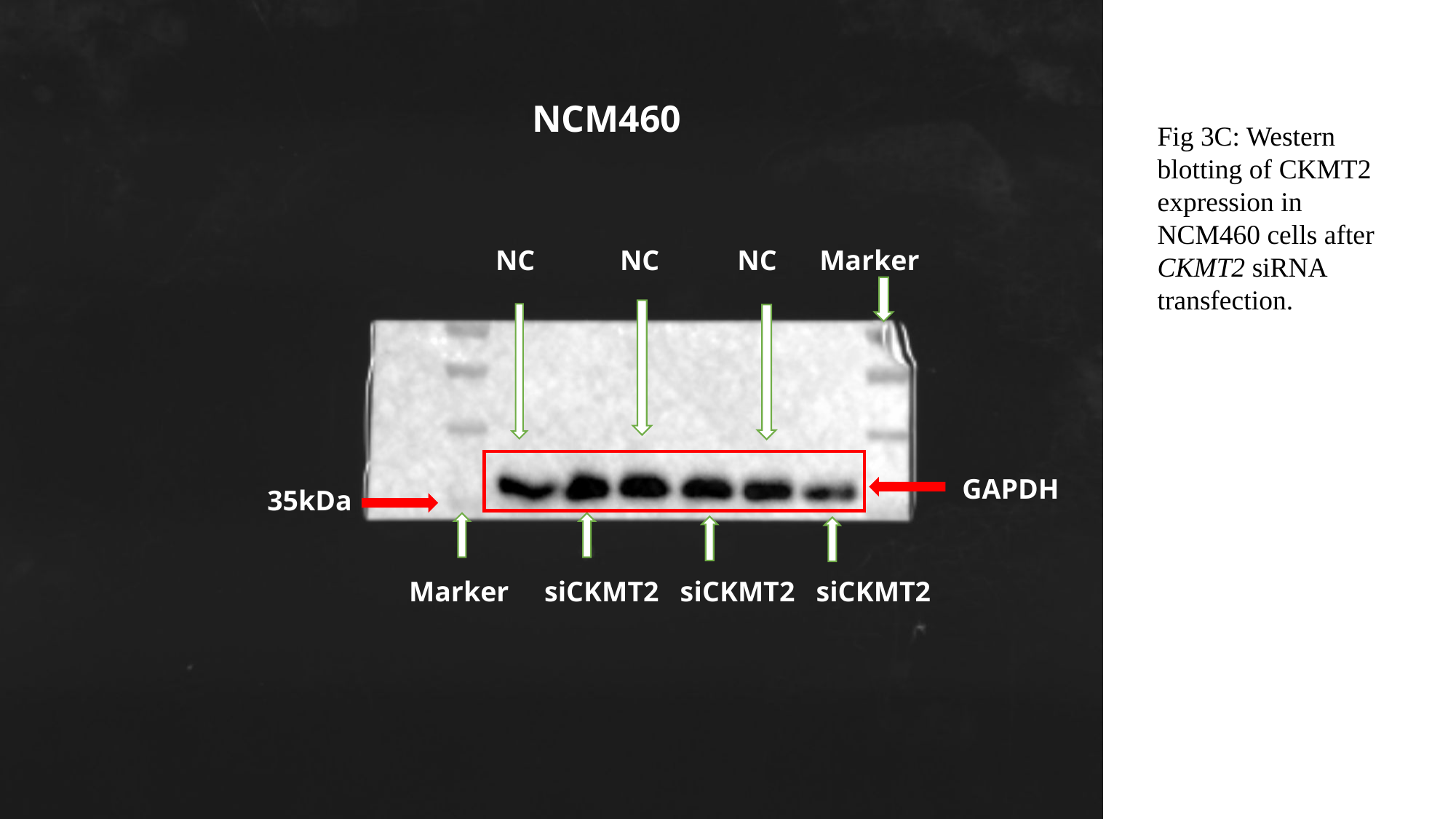

NCM460
Fig 3C: Western blotting of CKMT2 expression in NCM460 cells after CKMT2 siRNA transfection.
 NC NC NC Marker
GAPDH
35kDa
 Marker siCKMT2 siCKMT2 siCKMT2

## Slide 3
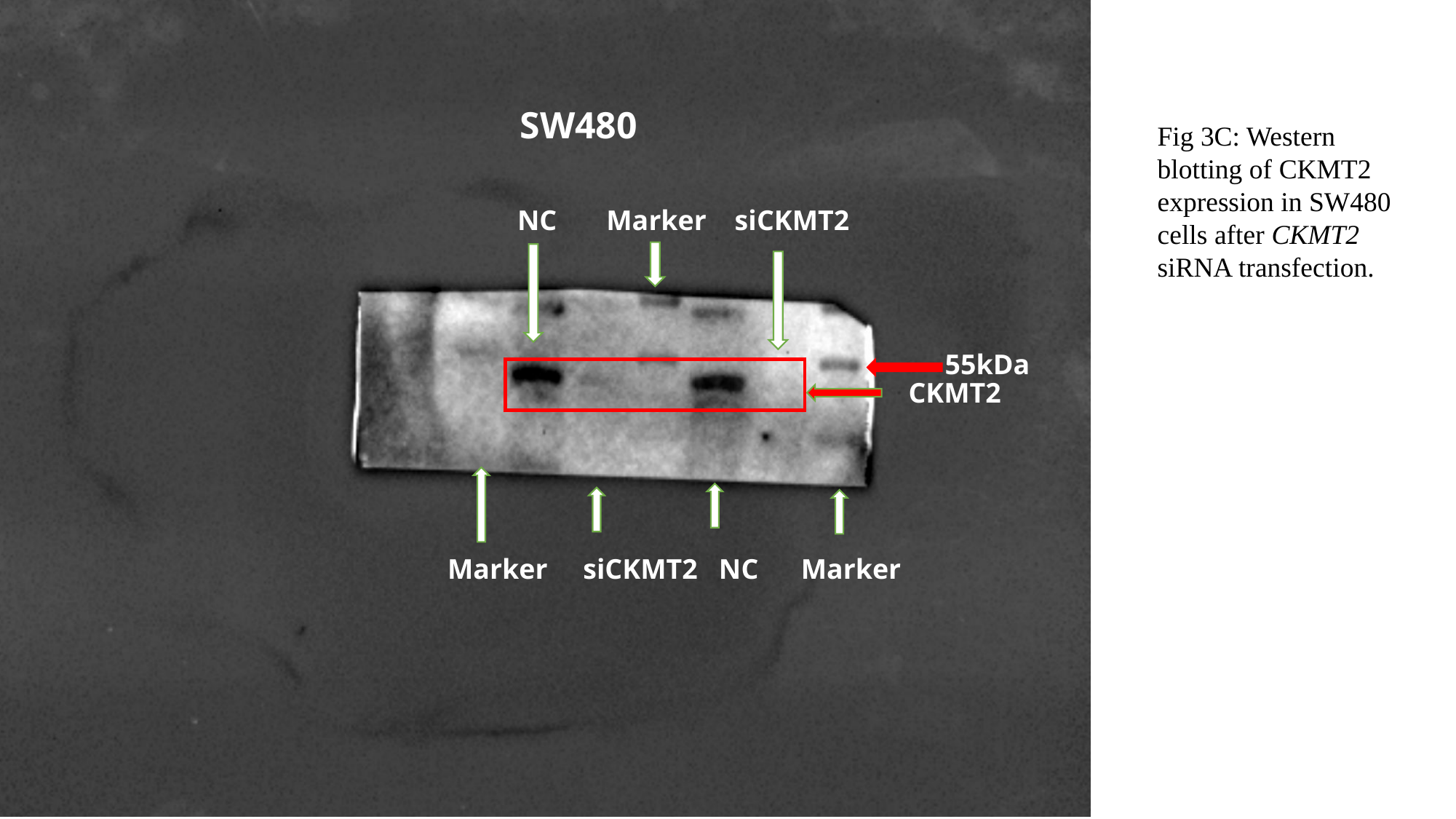

SW480
Fig 3C: Western blotting of CKMT2 expression in SW480 cells after CKMT2 siRNA transfection.
 NC Marker siCKMT2
55kDa
CKMT2
 Marker siCKMT2 NC Marker

## Slide 4
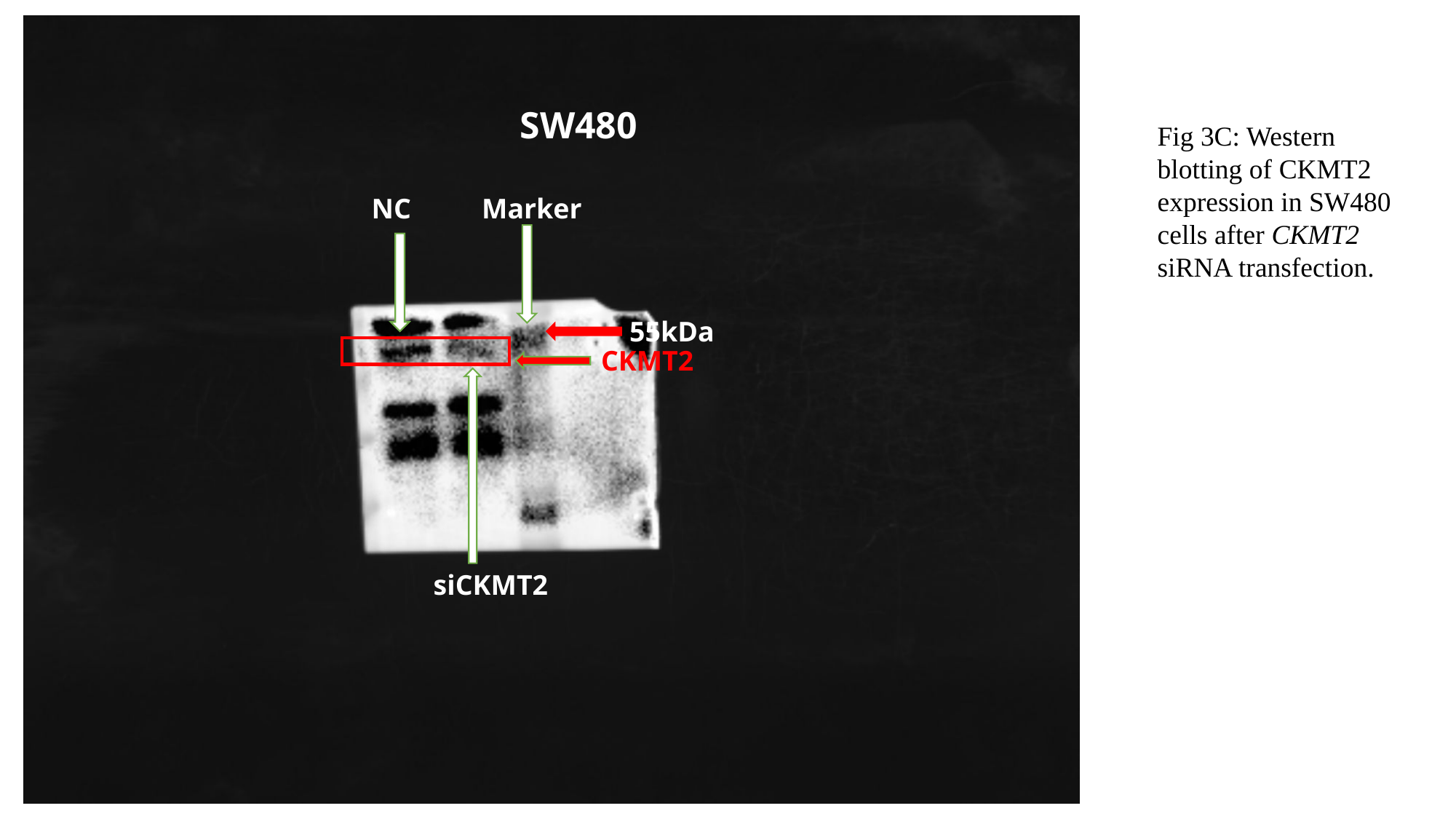

SW480
Fig 3C: Western blotting of CKMT2 expression in SW480 cells after CKMT2 siRNA transfection.
 NC Marker
55kDa
CKMT2
siCKMT2

## Slide 5
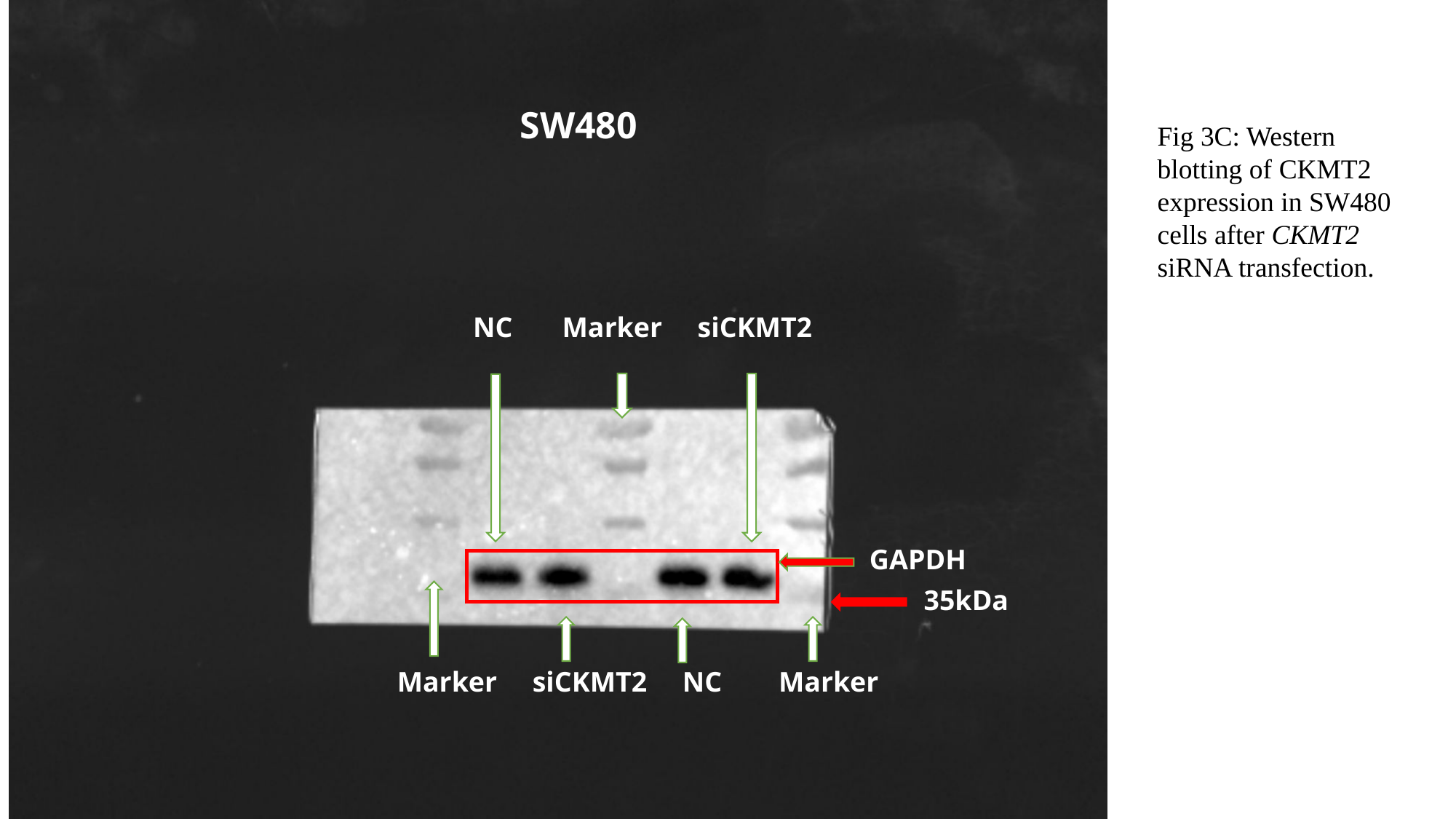

SW480
Fig 3C: Western blotting of CKMT2 expression in SW480 cells after CKMT2 siRNA transfection.
 NC Marker siCKMT2
GAPDH
35kDa
 Marker siCKMT2 NC Marker

## Slide 6
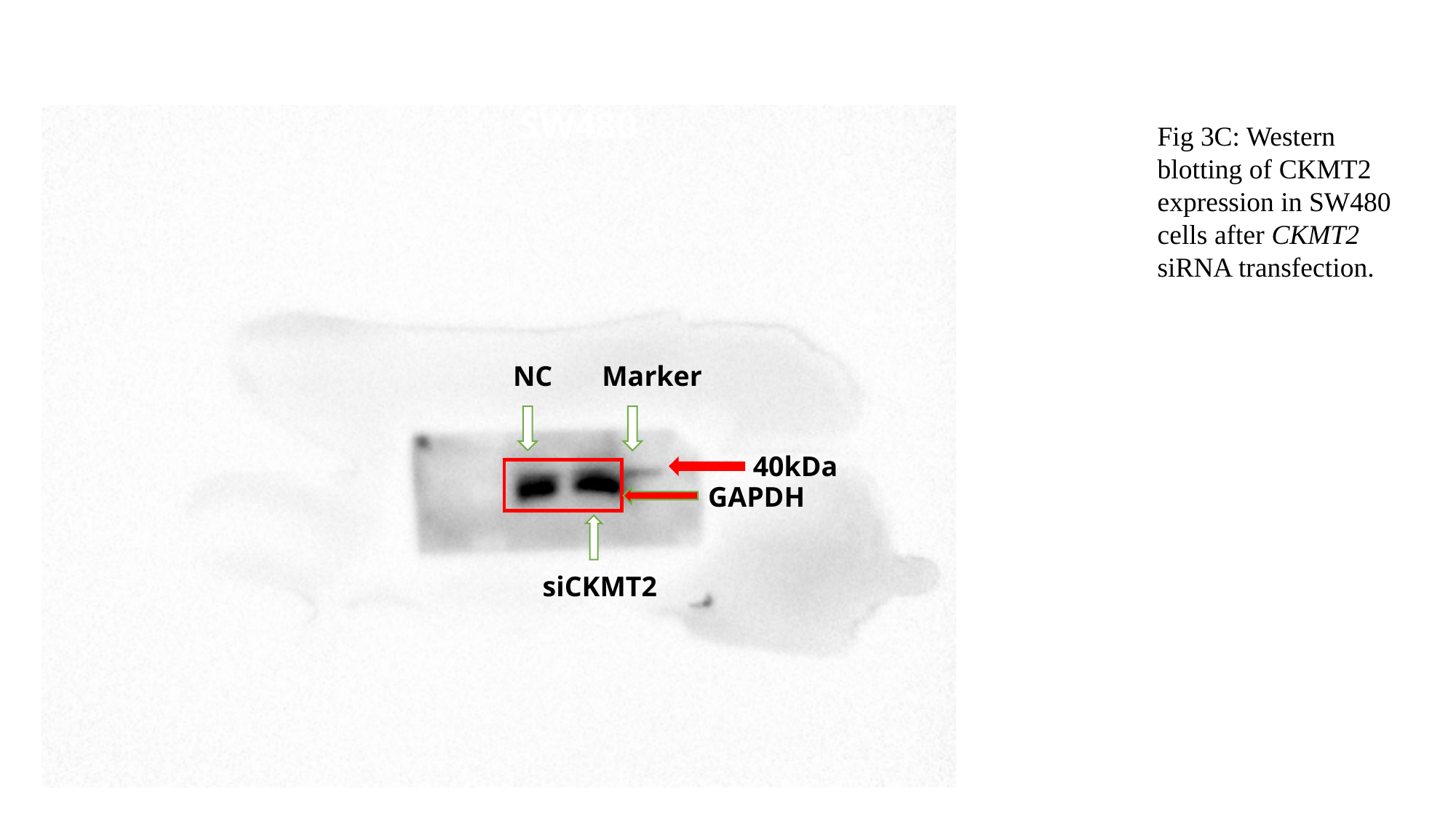

SW480
Fig 3C: Western blotting of CKMT2 expression in SW480 cells after CKMT2 siRNA transfection.
 NC Marker
40kDa
GAPDH
siCKMT2

## Slide 7
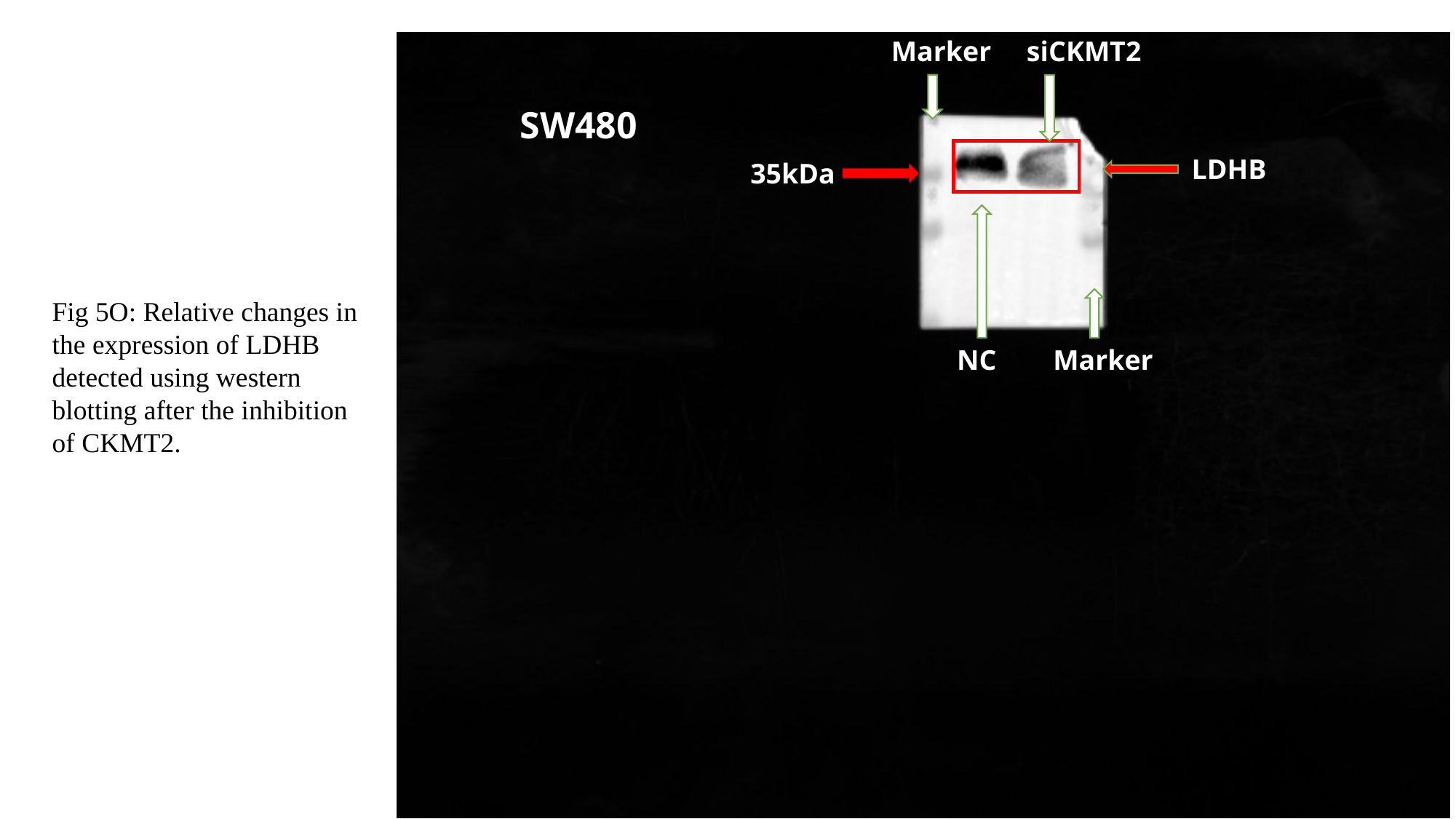

Marker siCKMT2
SW480
LDHB
35kDa
Fig 5O: Relative changes in the expression of LDHB detected using western blotting after the inhibition of CKMT2.
NC Marker

## Slide 8
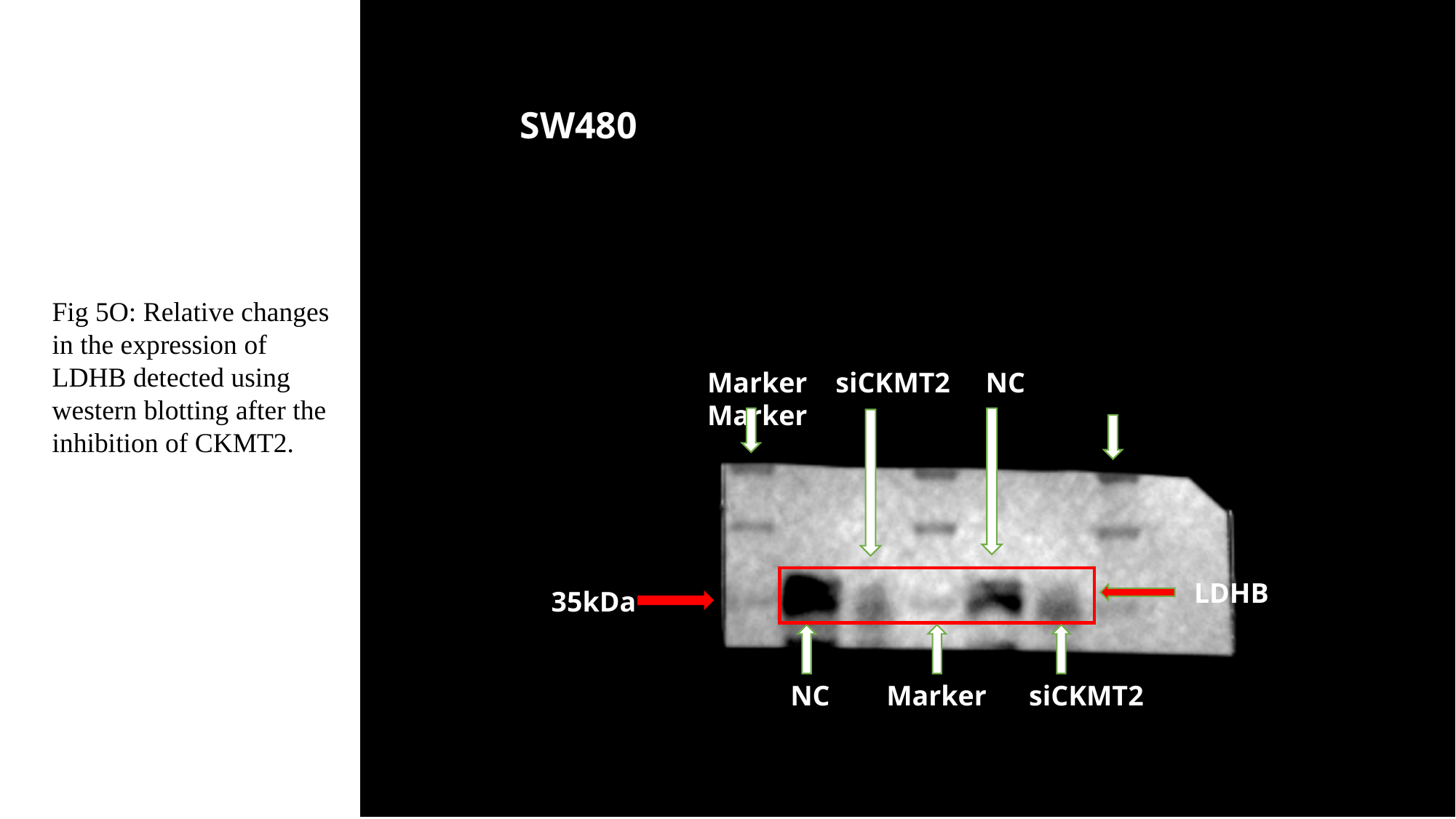

SW480
Fig 5O: Relative changes in the expression of LDHB detected using western blotting after the inhibition of CKMT2.
Marker siCKMT2 NC Marker
LDHB
35kDa
NC Marker siCKMT2

## Slide 9
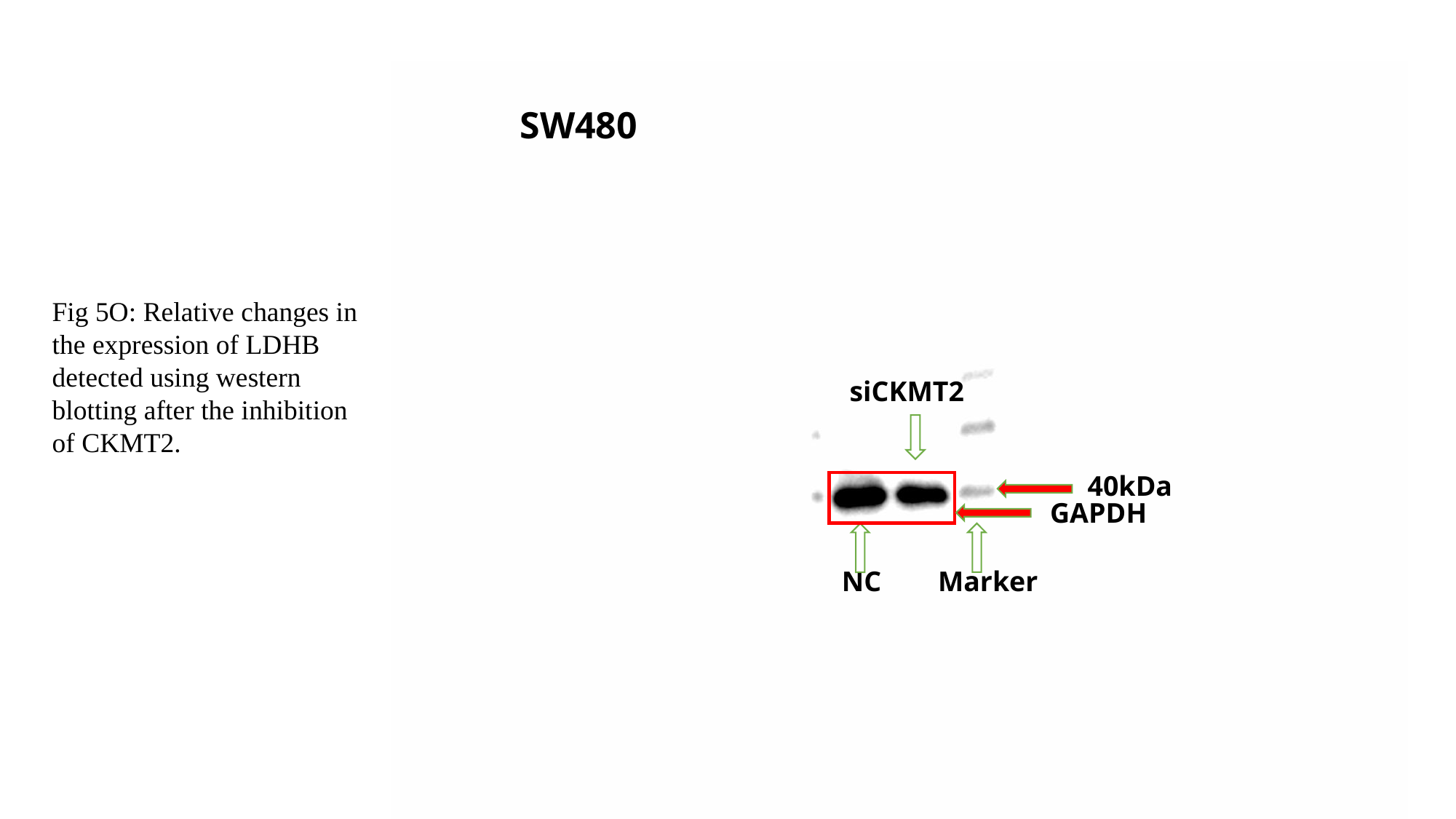

SW480
Fig 5O: Relative changes in the expression of LDHB detected using western blotting after the inhibition of CKMT2.
siCKMT2
40kDa
GAPDH
NC Marker

## Slide 10
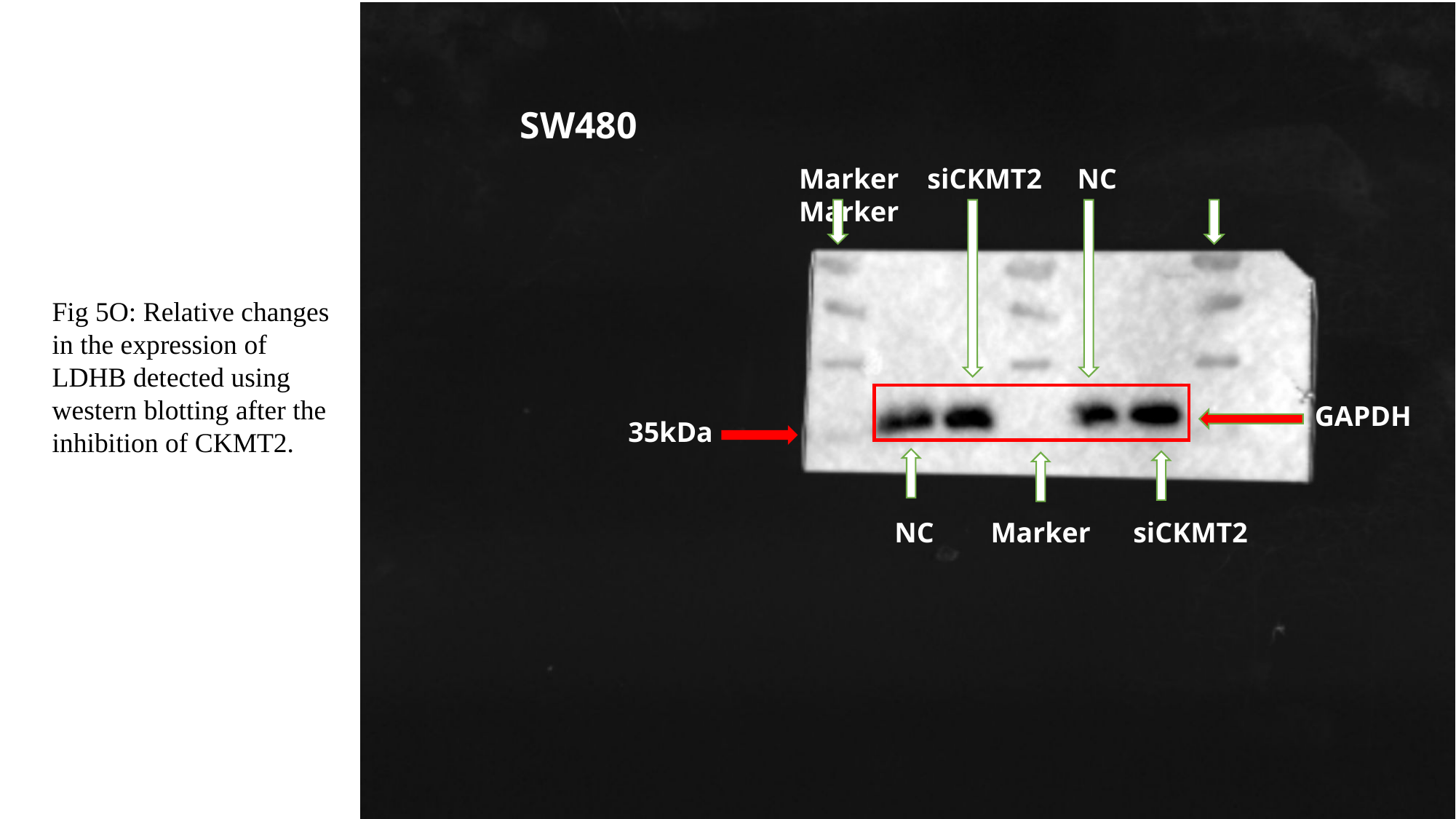

SW480
Marker siCKMT2 NC Marker
Fig 5O: Relative changes in the expression of LDHB detected using western blotting after the inhibition of CKMT2.
GAPDH
35kDa
 NC Marker siCKMT2

## Slide 11
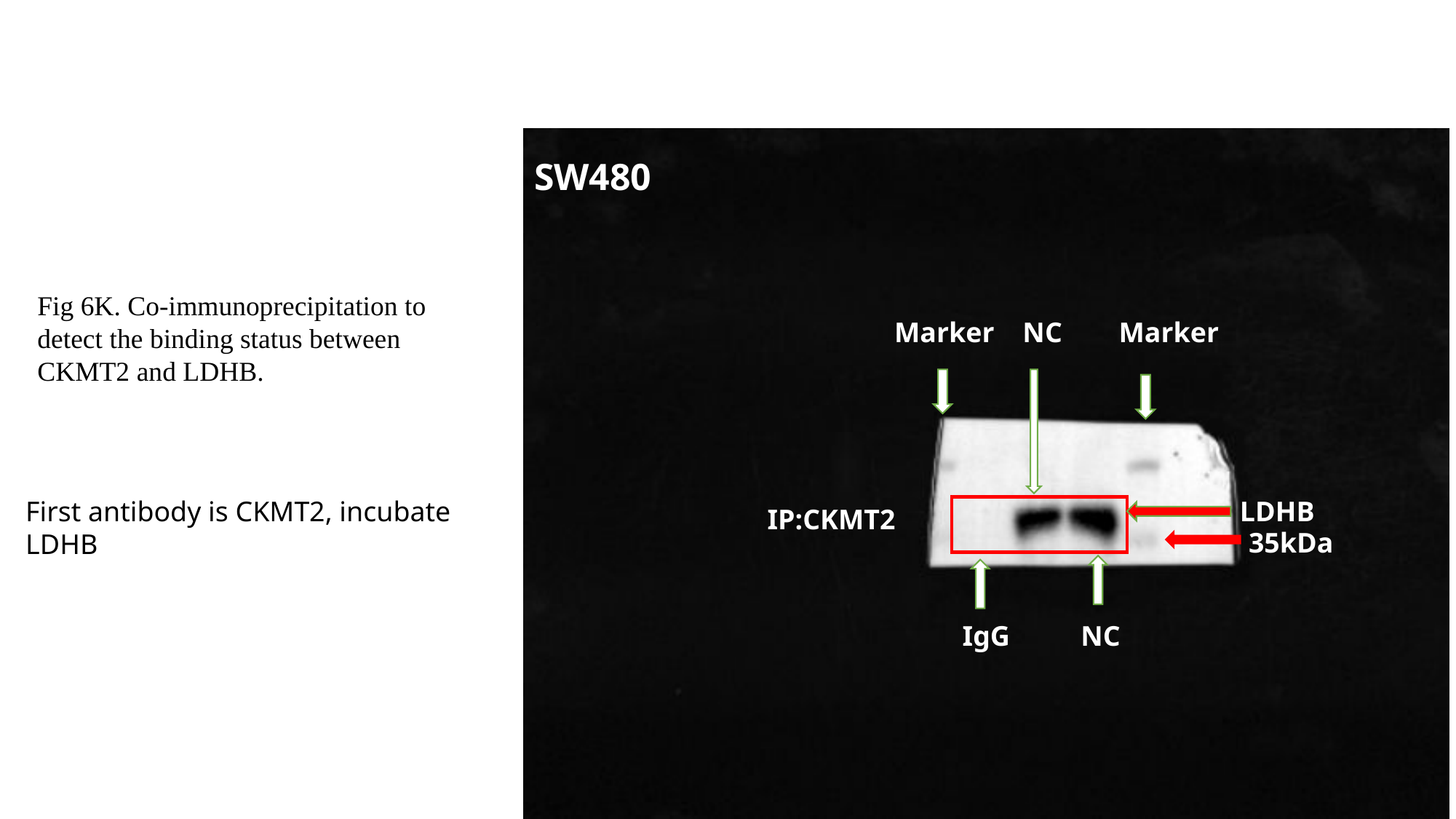

SW480
Fig 6K. Co-immunoprecipitation to detect the binding status between CKMT2 and LDHB.
Marker NC Marker
First antibody is CKMT2, incubate LDHB
LDHB
IP:CKMT2
35kDa
 IgG NC

## Slide 12
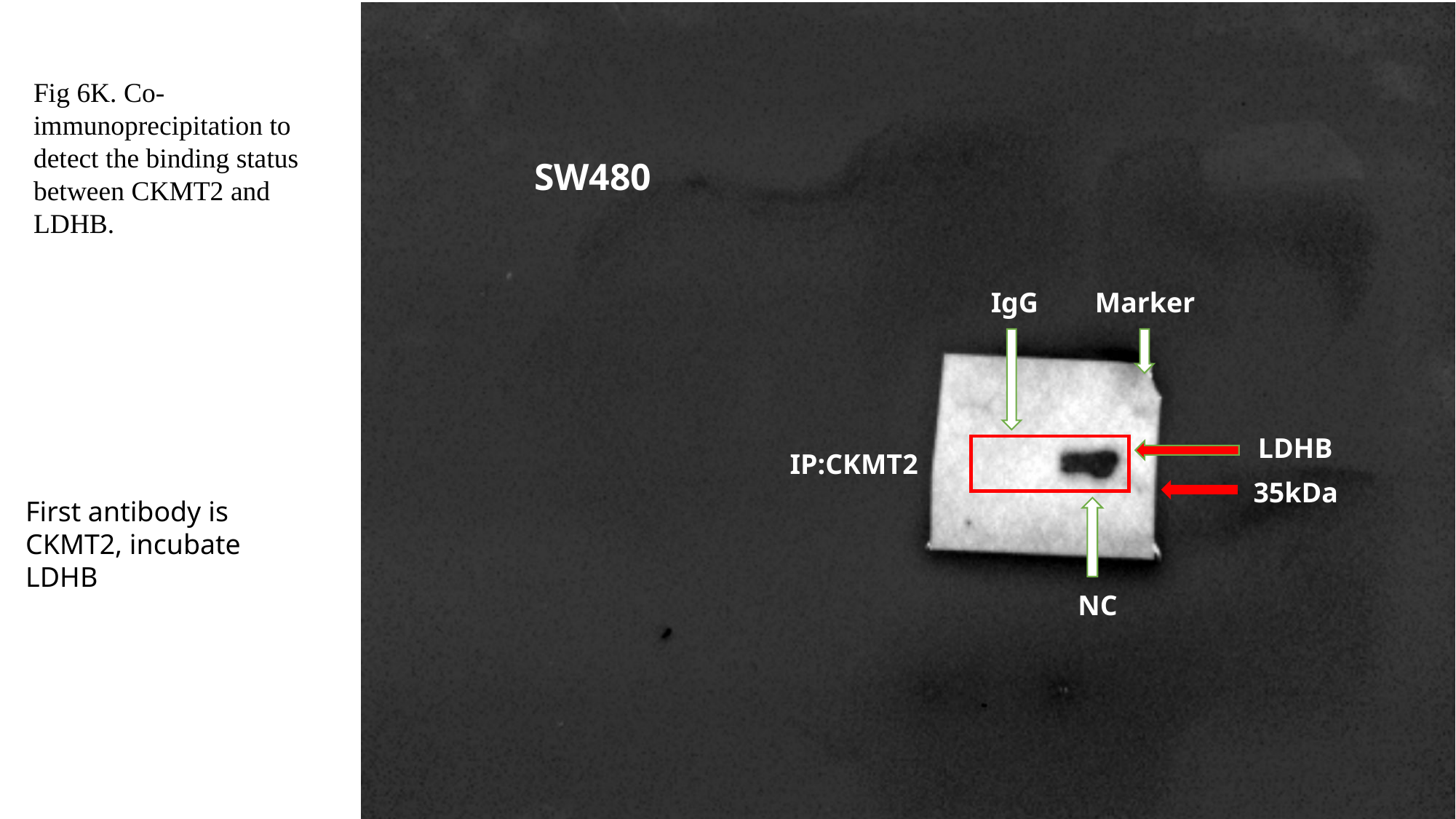

Fig 6K. Co-immunoprecipitation to detect the binding status between CKMT2 and LDHB.
SW480
IgG Marker
LDHB
IP:CKMT2
35kDa
First antibody is CKMT2, incubate LDHB
NC

## Slide 13
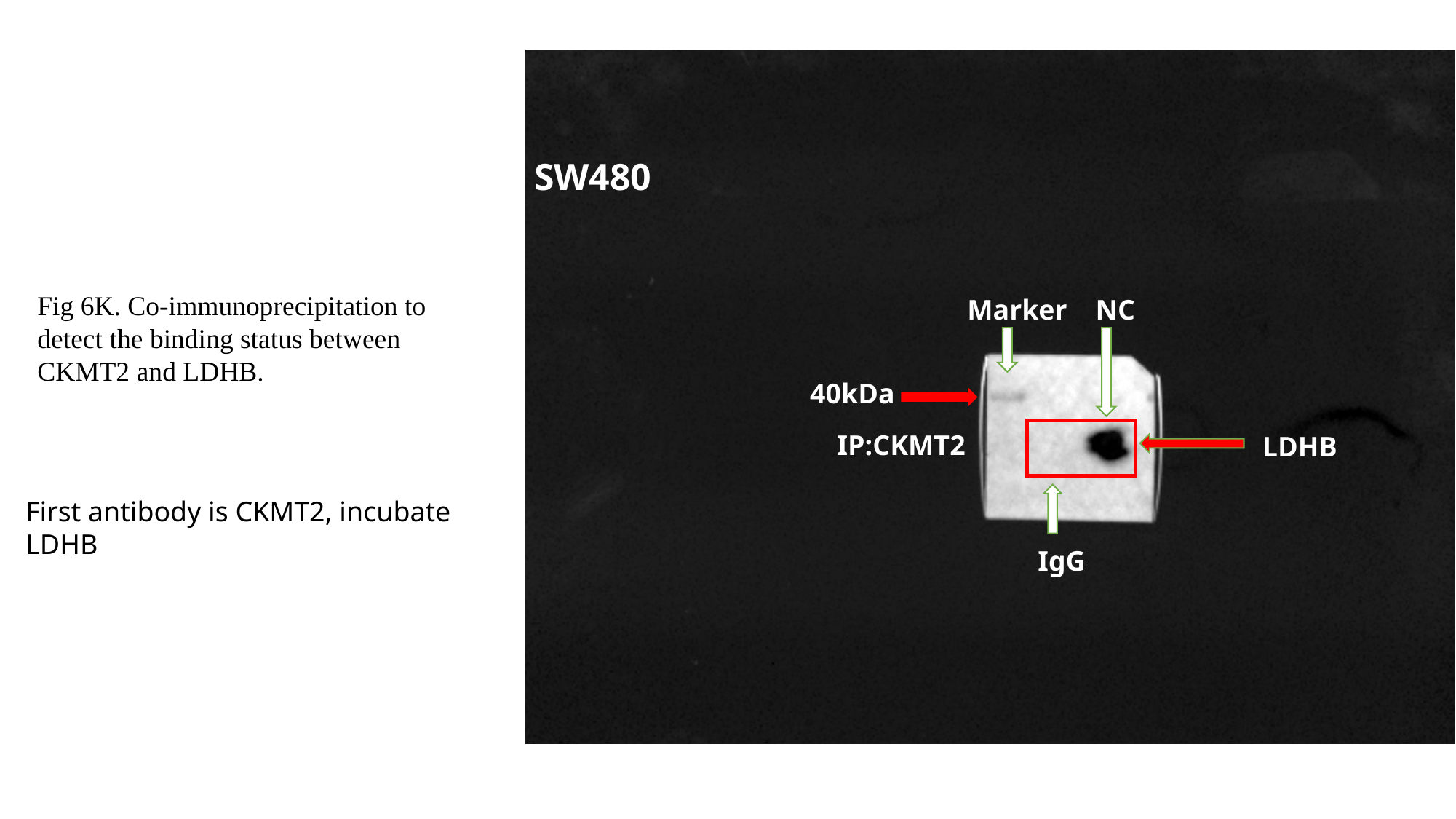

SW480
Fig 6K. Co-immunoprecipitation to detect the binding status between CKMT2 and LDHB.
Marker NC
40kDa
IP:CKMT2
LDHB
First antibody is CKMT2, incubate LDHB
 IgG

## Slide 14
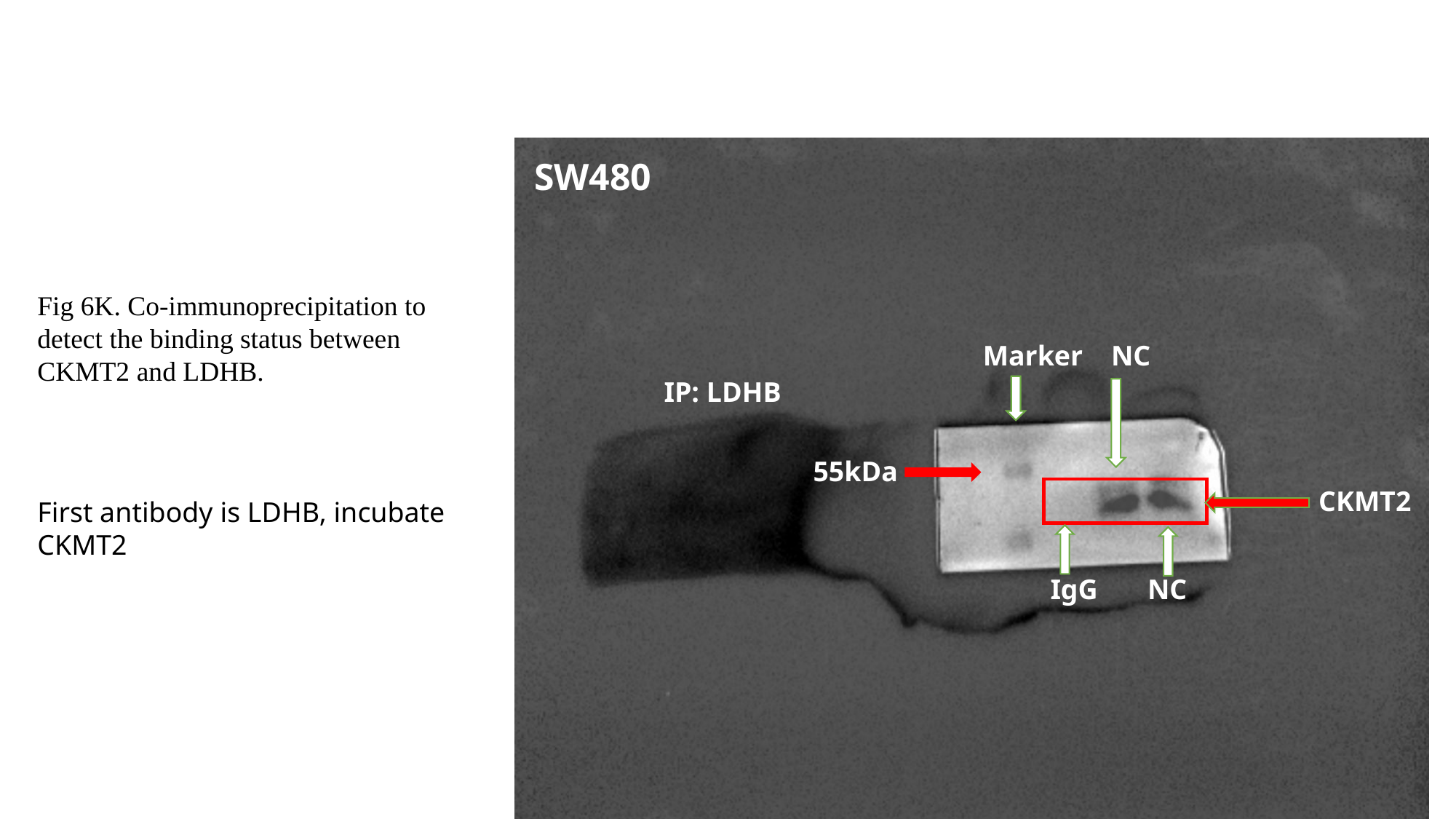

SW480
Fig 6K. Co-immunoprecipitation to detect the binding status between CKMT2 and LDHB.
Marker NC
IP: LDHB
55kDa
CKMT2
First antibody is LDHB, incubate CKMT2
 IgG NC

## Slide 15
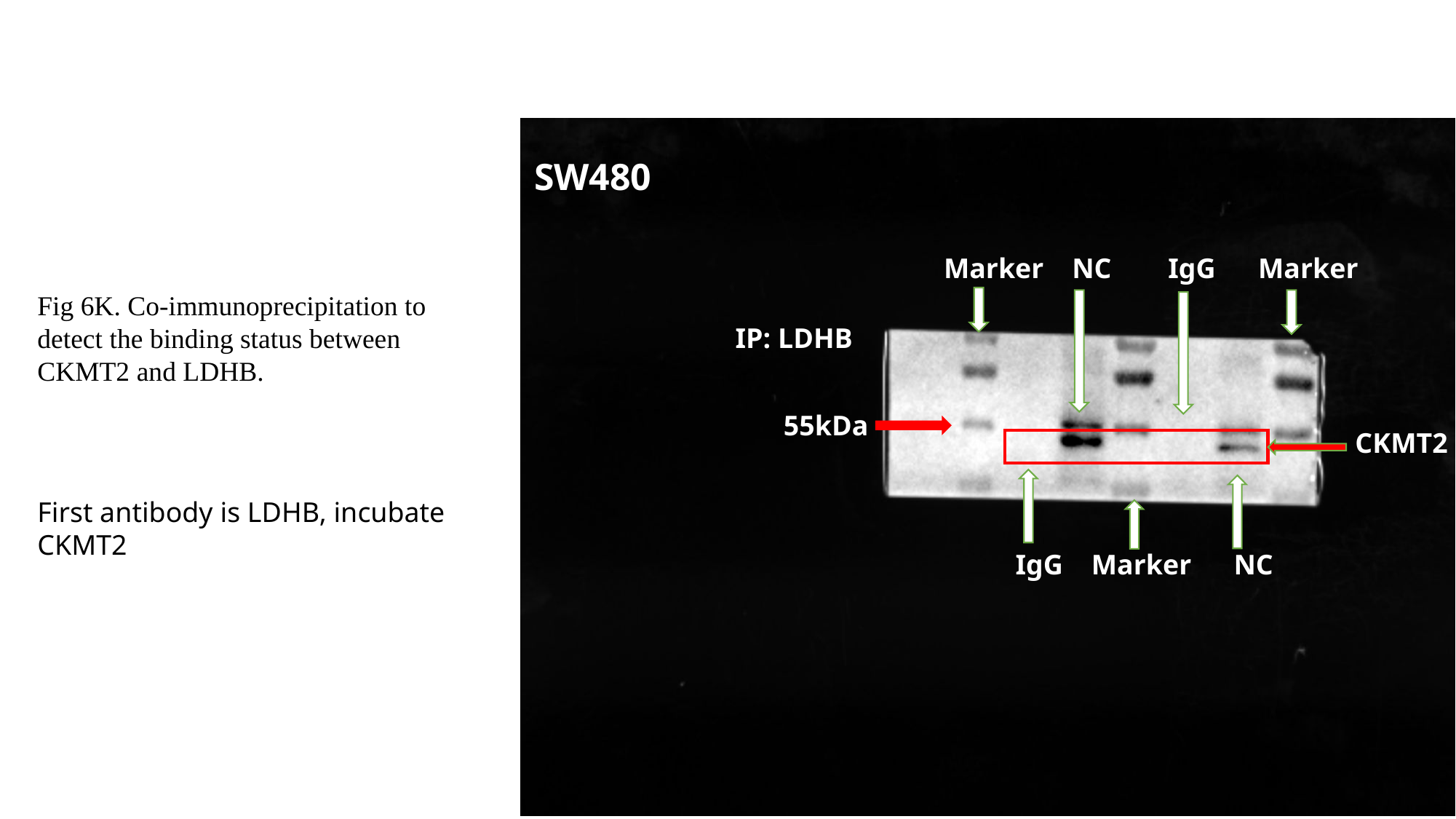

SW480
Marker NC IgG Marker
Fig 6K. Co-immunoprecipitation to detect the binding status between CKMT2 and LDHB.
IP: LDHB
55kDa
CKMT2
First antibody is LDHB, incubate CKMT2
IgG Marker NC

## Slide 16
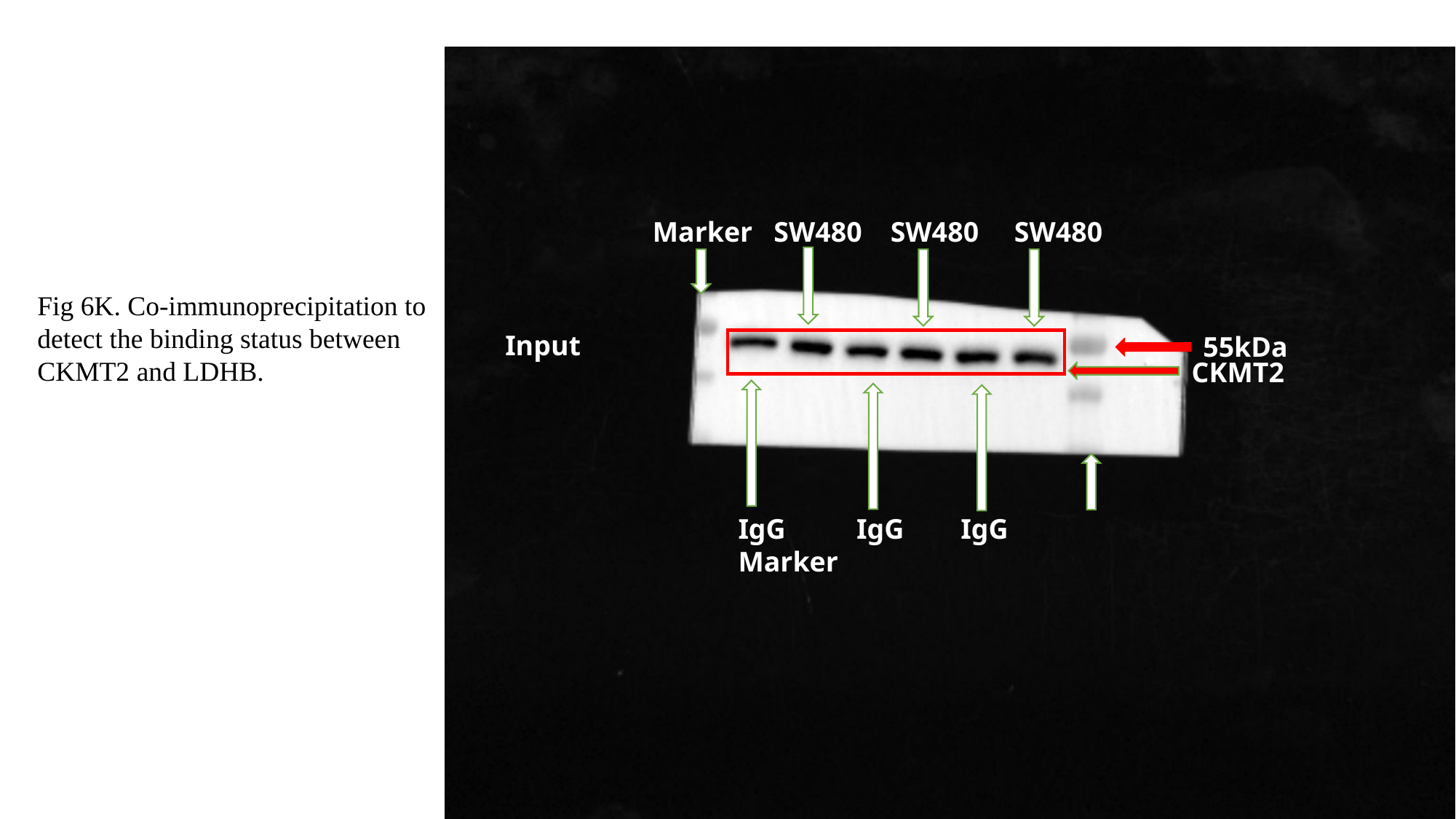

Marker SW480 SW480 SW480
Fig 6K. Co-immunoprecipitation to detect the binding status between CKMT2 and LDHB.
Input
55kDa
CKMT2
IgG IgG IgG Marker

## Slide 17
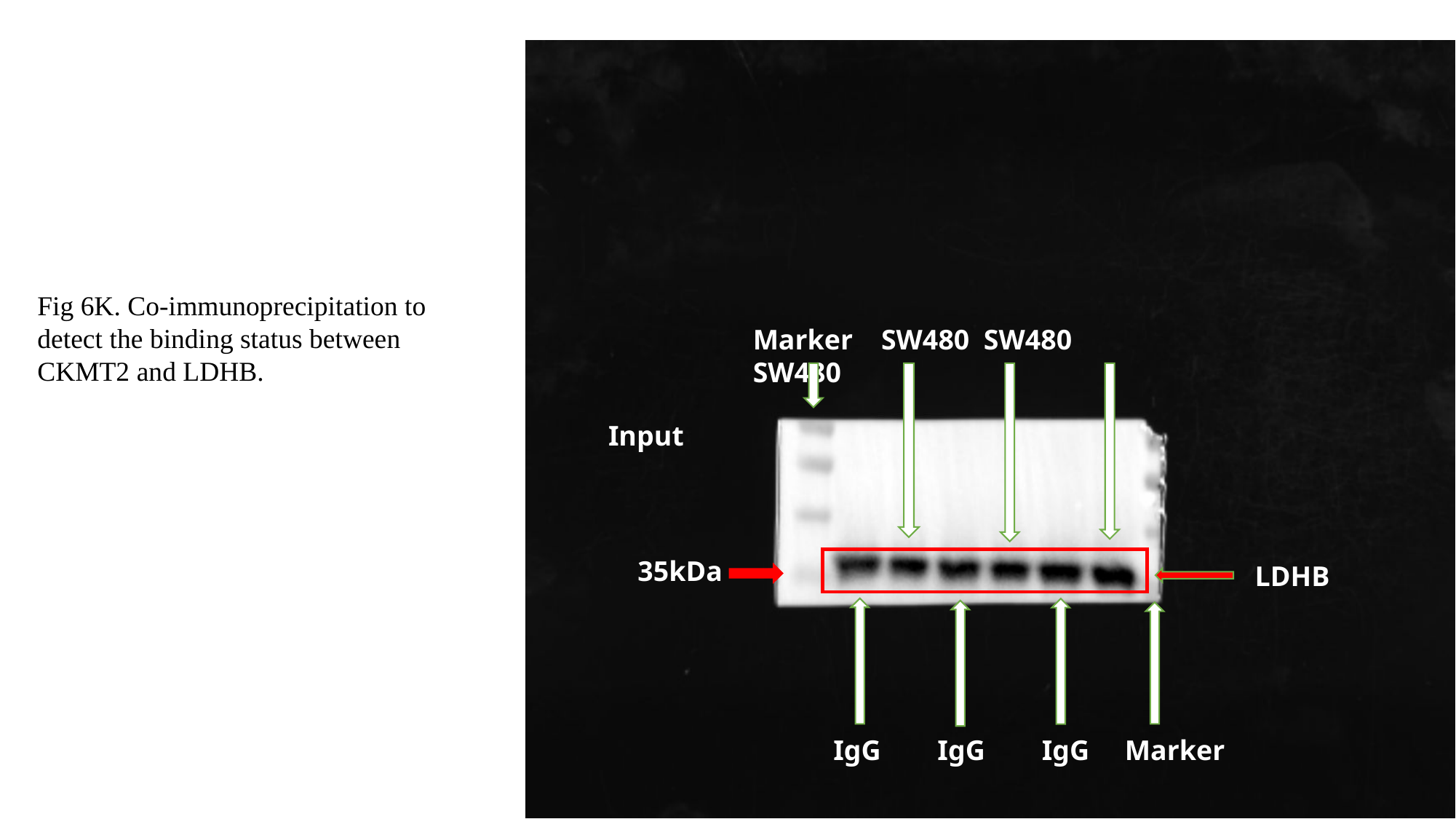

Fig 6K. Co-immunoprecipitation to detect the binding status between CKMT2 and LDHB.
Marker SW480 SW480 SW480
Input
35kDa
LDHB
IgG IgG IgG Marker

## Slide 18
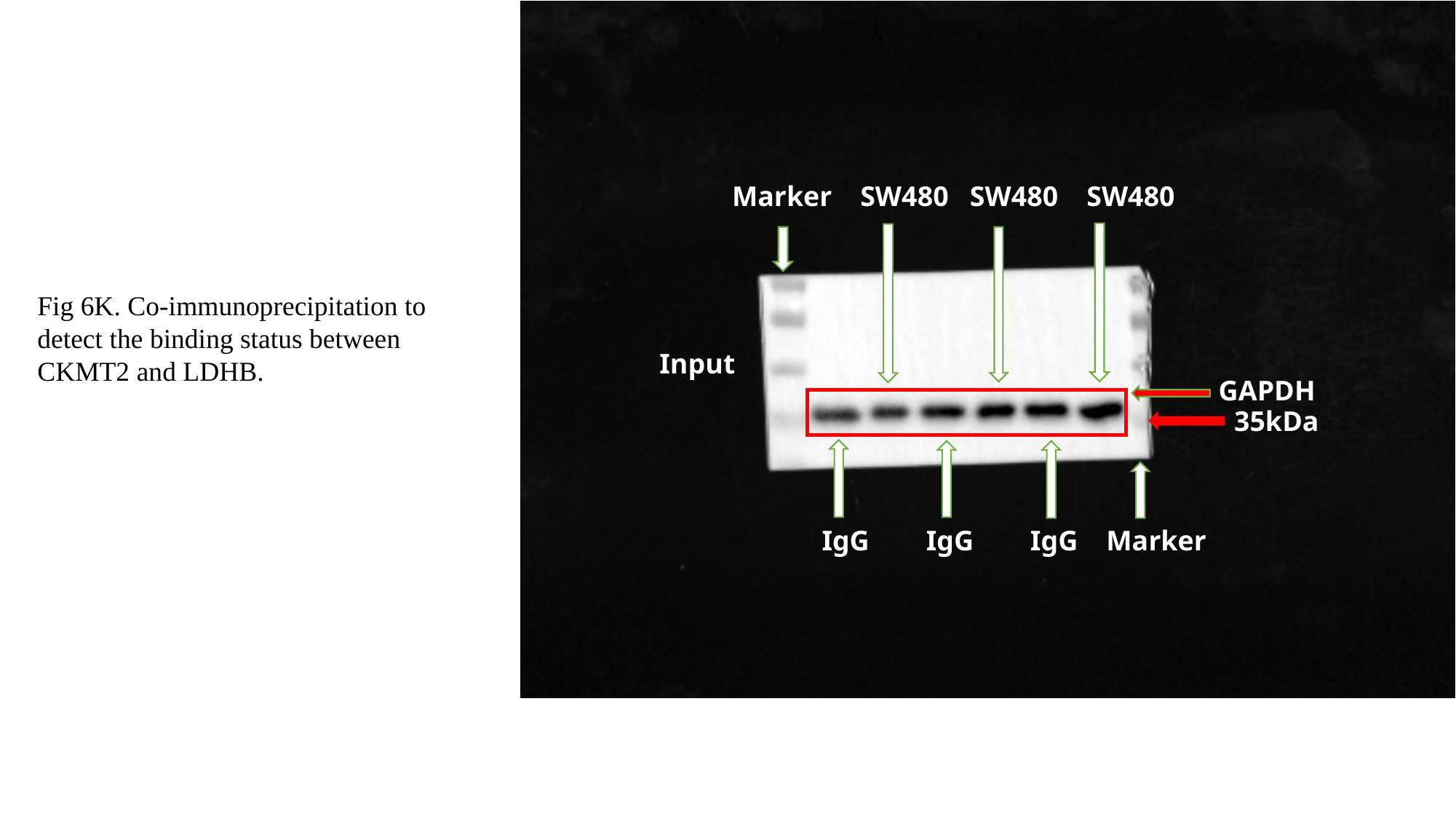

Marker SW480 SW480 SW480
Fig 6K. Co-immunoprecipitation to detect the binding status between CKMT2 and LDHB.
Input
GAPDH
35kDa
IgG IgG IgG Marker
